# Supplementary material for: High-resolution separation of bioisomers using ion cloud profiling
Source: Nat Commun. 2023 Mar 20;14:1535. doi: 10.1038/s41467-023-37281-7 (PMC10027677; doi:10.1038/s41467-023-37281-7)
Supplement: Supplementary file 1 — Supplementary Information [file 41467_2023_37281_MOESM1_ESM.pdf]

High-Resolution Separation of Bioisomers Using Ion Cloud Profiling

Xiaoyu Zhou[1,2], Zhuofan Wang[1], Jingjin Fan[1], Zheng Ouyang[1,2]*

[1] State Key Laboratory of Precision Measurement Technology and Instruments, Department of Precision Instrument, Tsinghua University, Beijing 100084, China.
[2]Institute for Precision Medicine, Tsinghua University, Beijing 100084, China.

*Correspondence to: ouyang@tsinghua.edu.cn

Supplementary Information

This PDF file includes:

Supplementary Text
Supplementary Figures 1 to 10
Supplementary Tables 1 to 3
References 1-20

Supplementary Note 1

Performance of the typical ion mobility analysis

The importance of distinguishing bioisomers is well recognized for studies in chemistry and life sciences; the analysis of bioisomers has remained as a challenge even for state-of-the-art ion mobility mass spectrometers (Supplementary Fig. 1). In earlier setups, as Synapt G2 (Waters, Wilmslow, U.K.) and tims-TOF (Bruker Daltonics, Bremen, Germany), their typical performances with ion mobility resolution of 40 to 300 were always inadequate for analysis of bioisomers, such as glycan (Supplementary Fig. 1a) [1] and lipid isomers (Supplementary Fig. 1b) [2]. In recent years, more complexed configurations were employed to improve the ion mobility resolution. For instance, cyclic analyzer, as in Cyclic IMS (Waters, Wilmslow, U.K.), has a circulated ion mobility path, up to 100 cycles with a separation length of 98 m, which enables an ion mobility resolution of 750 for analyzing peptide isomers (Supplementary Fig. 1c) [3]. Structure for lossless ion manipulation (SLIM), developed by Pacific Northwest National Laboratory (PNNL), employs a 4-layer configuration, which enables a resolution of 561 for analyzing peptide isomers under a separation length of 43.2 m (Supplementary Fig. 1d) [4]. With an extended ion separation path up to 1094 m, a higher ion mobility resolution of 1860 was claimed for analyzing Agilent tuning mixture (not isomers) [5], but has not been shown for analyzing bioisomers.

In this work, ion cloud profiling method operated with a LIT was used to allow high-resolution separations of over 10,000 for analysis of bioisomers (Supplementary Fig. 1e). The LIT has a simple configuration, which has been widely employed as an ion processing device in modern hybrid mass spectrometers. In future, the ion cloud profiling technology is expected to facilitate a broad range of applications for biological study.

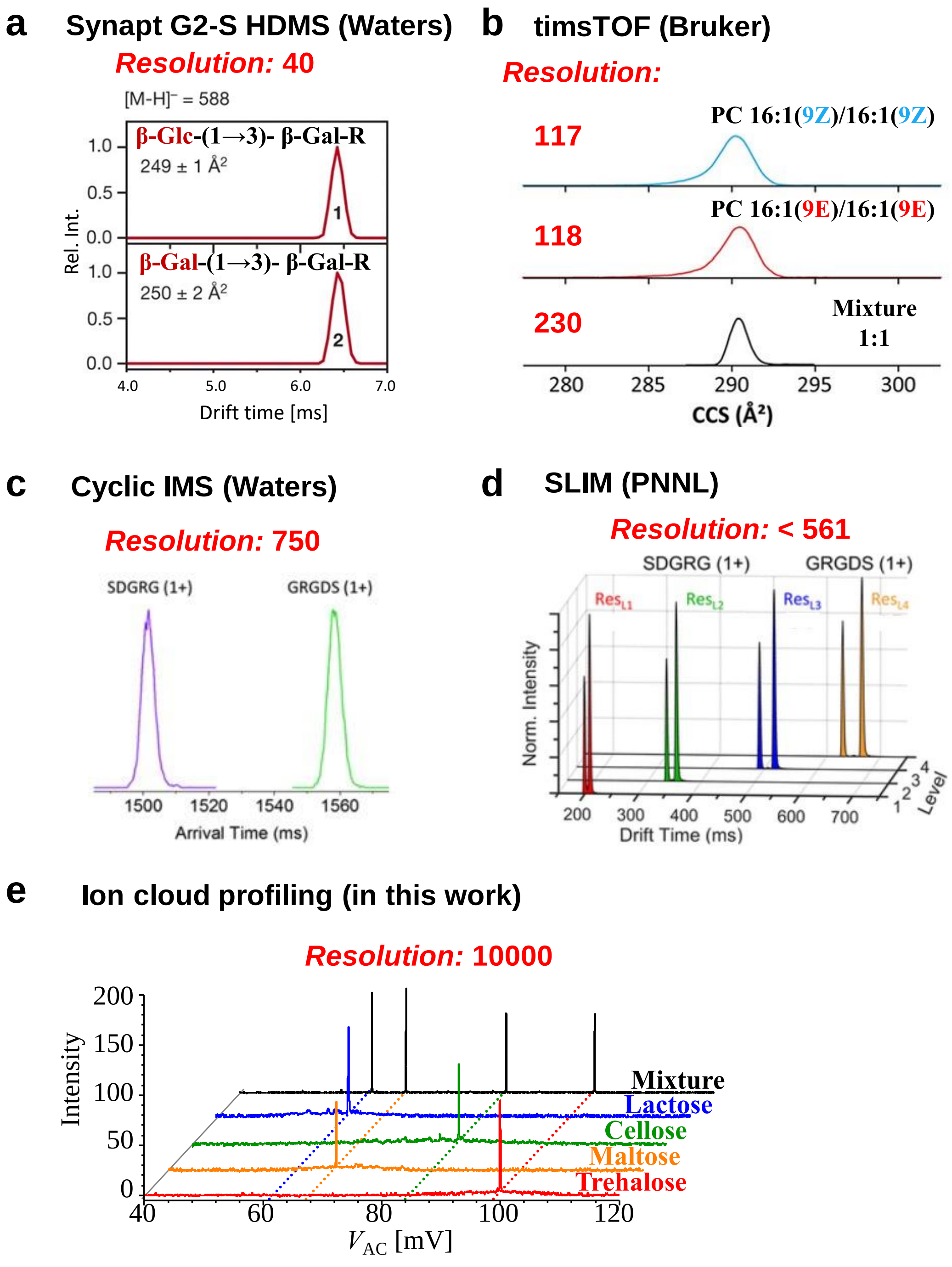

Supplementary Fig. 1. Bioisomer analysis using ion mobility. (**a**) Ion mobility spectra of two disaccharide isomers using Synapt G2 (Waters, Wilmslow, U.K.). Taken from ref (1) with permission from Springer Nature. (**b**) Ion mobility spectra of two PC isomers and their mixture using tims-TOF (Bruker Daltonics, Bremen, Germany). Taken from ref (2) with permission from American Chemical Society. (**c**) Ion mobility spectrum of two peptide isomers using Cyclic IMS (Waters, Wilmslow, U.K.). Taken from ref (3) with permission from American Chemical Society. (**d**) Ion mobility spectra of two peptide isomers using SLIM (Pacific Northwest National Laboratory, PNNL).

Taken from ref (4) with permission from American Chemical Society. (**e**) Profiling spectrum of four disaccharide isomers in this work.

Supplementary Note 2
Ion cloud profiling method

Ion cloud profiling was developed for analysis of the isomeric ions. Isomeric ions have identical mass and thus the identical resonance frequency of the AC [6]. Fig. 1c shows at resonance, two isomeric ions with different damping coefficients, b = 0.0010 (blue) and 0.0012 (purple), were separated by their ion trajectories. In addition, the physical feature of resonance reveals that in an ideal case, the ion trajectories of one ion species should ultimately achieve an identical ion trajectory, regardless the initial condition of the ions (Supplementary Fig. 2). For instance, without resonance excitation, two isomeric ion species were cooled to the trap center with a relatively loose ion cloud (Supplementary Fig. 2a). Then, they were separated into two compact ion packs subjected to the resonance (Supplementary Fig. 2b), which indicated a possible way for structural analysis of isomers.

For experimental implementation, the complete ion cloud profiling method consists of sample ionization using nESI, ion introduction through DAPI, selection of isomeric ions in LIT I, and structural analysis in LIT II. Isomeric ions generated by nESI at atmospheric pressure were introduced into the vacuum via the DAPI. Through resonance excitation, isomeric ions were mass selectively transferred from LIT I to LIT II, where matrix interference of different mass of the isomers were removed. Isomeric ions after mass selection were trapped and cooled at Mathieu parameter, q = 0.4 in LIT II for 600 ms. For structural analysis of the isomers, the ions were subject to the resonance excitation by scanning the AC amplitude linearly. When the ion amplitude exceeded the trap boundary, they were ejected out of the trap and detected by the detector. The DC and RF/AC controls used for structural analysis of the isomers are summarized in Supplementary Tables 2 and 3.

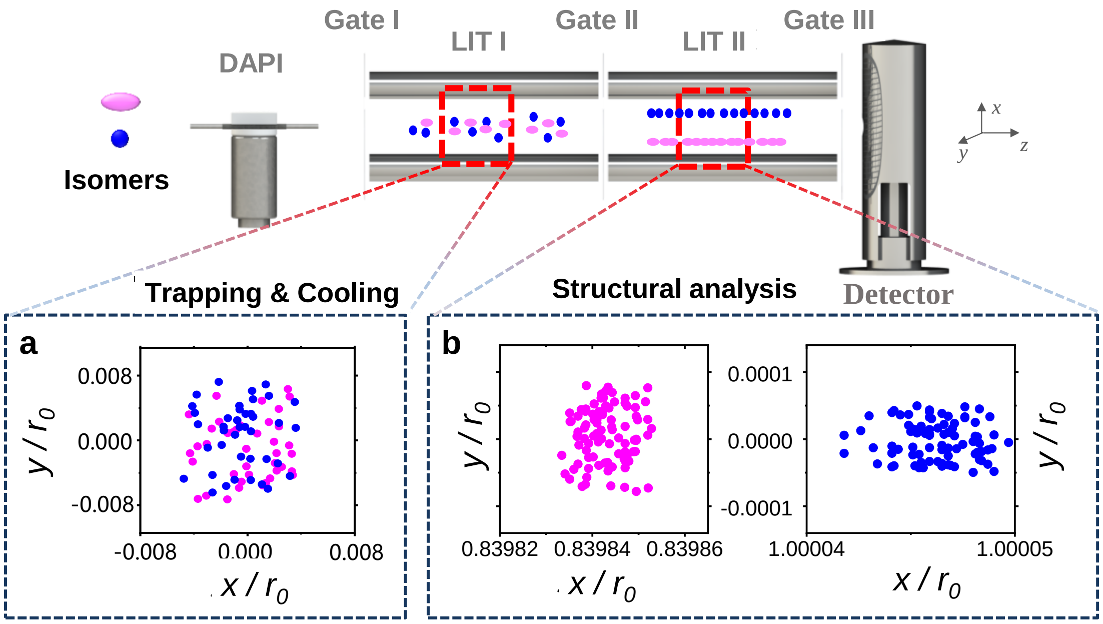

Supplementary Fig. 2. Simulated ion clouds of the isomers. Simulated ion clouds for (**a**) ion trapping and cooling in LIT I and (**b**) ion cloud profiling for structural analysis in LIT II. Two isomeric ions were used for simulation with damping coefficients: b = 0.001 (blue) and 0.0012 (purple). During structural analysis, the two isomeric ion species, which are mixed in ion trapping and cooling, become separated and ejected sequentially to the detector.

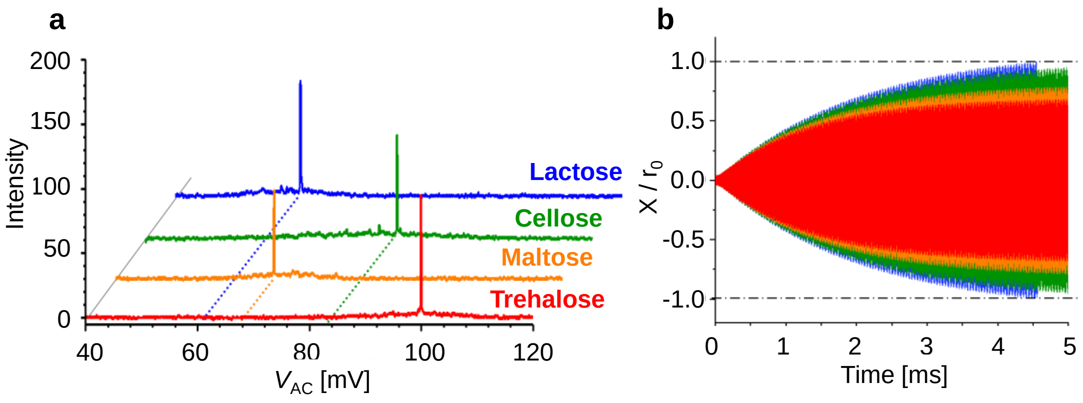

Supplementary Fig. 3. Experiments and simulation of four disaccharides isomers. **(a)** Ion cloud profiling spectra of the four disaccharides: trehalose (red), maltose (orange), cellose (green), lactose (blue). **(b)** Simulated ion trajectories for four isomeric ions characterized by the reduced damping coefficients: $b'_{lactose}$ = 0.001875 (blue), $b'_{cellose}$ = 0.002053 (green), $b'_{maltose}$ = 0.002556 (orange) and $b'_{trehalose}$ = 0.003004

(red). Here, $b^{'} = 2b/\Omega m$, where Ω is the angular frequence of the RF field, m is ion mass, b is the damping coefficient of the ions.

Supplementary Table 1. The key properties of our ion mobility platform and the commercial instruments.

Instruments		Measurable quantities	Calibration required	Resolving powers ($\Omega/\Delta\Omega$)	Acquisition per measurement	Time for a full spectrum
**Drift tube IMS**	**Atmospheric pressure**	Mobility and CCS (Ω)	No (reference standard)	250 [7]	Full spectrum	Milliseconds
	**Low pressure**	Mobility and CCS(Ω); α(E/N)		140 [8]	Single point	
**Cyclotron**		Mobility and CCS (Ω)	No	1040 [9]	Full spectrum for n = 1 Partial spectrum for n > 1	Seconds to minutes
**Cyclic-/SLIM-TW-IMS**		Mobility and CCS (Ω)	Yes	1860 [5]	Full spectrum	Milliseconds to seconds
**Trapped IMS**		Mobility and CCS (Ω)	Yes	400 [10]	Single point	Milliseconds to seconds
**FAIMS**		α(E/N)	No ion mobility measurement	460 in $CV/\Delta CV$ [11]	Full spectrum	Seconds to minutes
**Ion cloud profiling technology**		DCS	No typical CCS measurement	10000 in $V_{AC}/\Delta V_{AC}$	Full spectrum	Seconds

Supplementary Table 2. Scan function of DC voltages used for ion manipulation and structural analysis of the isomers (Unit [V]). The table shows the DC voltages applied on the ion source, gates I, II, and III, LITs I and II, and detector for ionization, ion introduction, ion selection, ion transmission, and ion cloud profiling for structural analysis of bioisomers.

Steps	Ion source	Gate I	LIT 1 DC	Gate II	LIT 2 DC	Gate III	Detector
Sample ionization	1500	4.2	-0.3	30	0.4	0	0
Ion introduction	1500	4.2	-0.3	30	0.4	0	0
Ion selection	0	30	-0.3	30	0.4	0	0
Ion transmission	0	30	-0.3	-0.4	-0.5	30	0
Structural analysis	0	30	-0.3	42.4	0.4	3.4	-1500

Supplementary Table 3. Scan function of RF and AC voltages used for ion manipulation and structural analysis of the isomers (Unit [V]). The table shows the RF and AC voltages used for analysis of different bioisomers. RF voltage is used for ion trapping and ion cooling. AC voltage is used for mass selection and structural analysis of bioisomers. The scan time and pressure during the analysis process are also shown

Sample	RF [V]		AC [mV]		Scan time [ms]	Pressure [Torr]
	LIT 1	LIT 2	For mass selection	Scan for structural analysis		
Disaccharides	524	360	470	30~180	50	1.9×10^{-5}
Phospholipids	1091	750	255	55~110	100	1.9×10^{-5}
Peptide PTMs	798	548	387	40~95	80	1.9×10^{-5}

Supplementary Note 3

Performance characterization and optimization of the ion cloud profiling method

As a validation of the method, the assignments of each isomeric peaks were performed by identifying characteristic ion fragmentation patter (Supplementary Fig. 4) or adjusting the concentrations of the samples. For instance, some specific isomers, e.g., trehalose and lactose, m/z 365, have characteristic fragments, m/z 203 for trehalose and m/z 305, for lactose, which could be identified by tandem MS analysis (Supplementary Fig. 4). The characteristic fragments of trehalose and lactose were as in the literature [12]. For other isomers, the peaks could be identified by adjusting the peak intensity in the spectrum and concentrations of the samples.

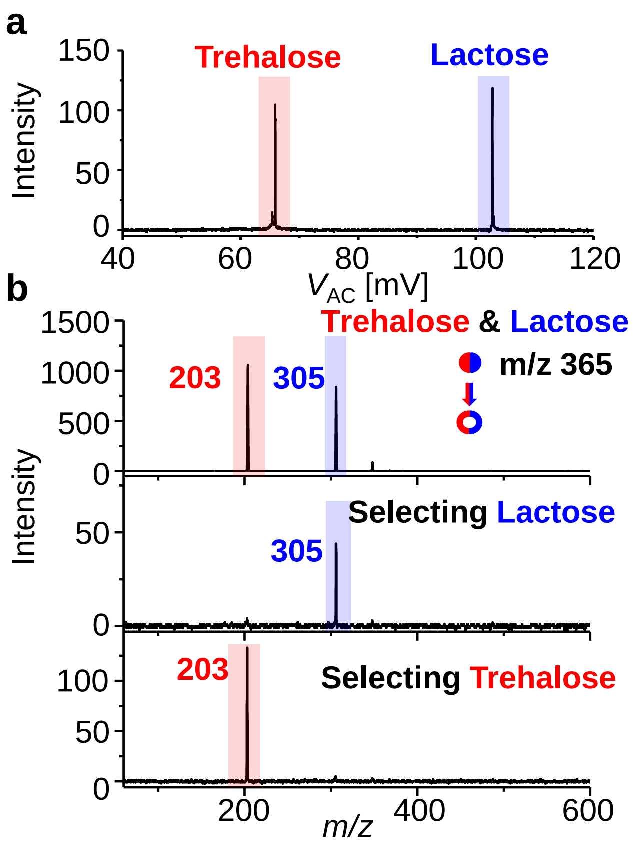

Supplementary Fig. 4. Structural analysis of disaccharide mixture. (**a**) Ion cloud profiling spectra of trehalose and lactose mixture. (**b**) Tandem MS spectra of lactose and trehalose mixture (top) and selected lactose (middle) and trehalose ions (bottom) from the mixture using ion cloud profiling. Trehalose (red) and lactose (blue) have characteristic fragments of 203 (red) and 305 (blue), respectively. Trap I was used to select and separate isomers to Trap II and Trap II was used for fragmentation of selected isomers. The excitation energies for ion fragmentation were 180 mV for mixture (top) and 148 mV for both selected lactose (middle) and trehalose ions (bottom), respectively.

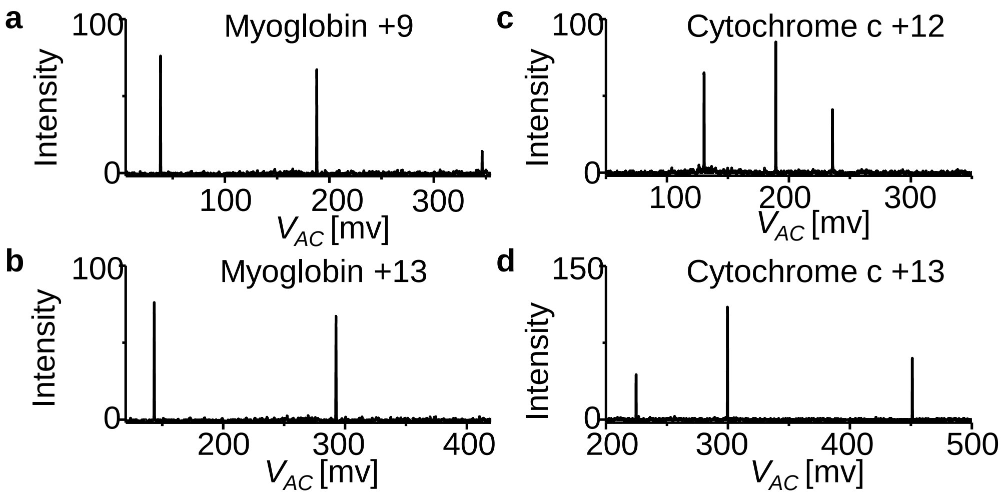

Supplementary Fig. 5. Ion cloud profiling spectra of protein with different charge state. Ion cloud profiling spectra of (**a**) myoglobin +9 charge state, (**b**) myoglobin +13 charge state, (**c**) cytochrome c +12 charge state, (**d**) cytochrome c +13 charge state. Multiple peaks represent different conformations of the protein ions. The results of the conformations of these protein ions are consistent with the previous literatures [13].

The performance of the ion cloud profiling method would be limited by non-ideal factors, such as the space charge effect due to the trapping of an excessive number of ions [14] and the dynamic RF effect [15]. Optimization of the working parameters in response to the non-ideal factors were discussed in below.

For space charge effect, it was observed that the increase of trapped ion number in LIT II led to a blue shift of V_{AC} to larger values (Supplementary Fig. 6). Meanwhile, the single peak of trehalose with ion number, PA = 43 mV (top), became split for larger PAs (middle and bottom, Supplementary Fig. 6a), where PA is the peak area of the ions in the spectrum. A similar phenomenon was also observed for the lactose and cellose mixture (Supplementary Fig. 6b). A possible explanation for the phenomenon was the nonlinear ion motion frequency shift due to the space charge effect [14,16], which resulted in the simultaneous shift of V_{AC} and peaking spitting.

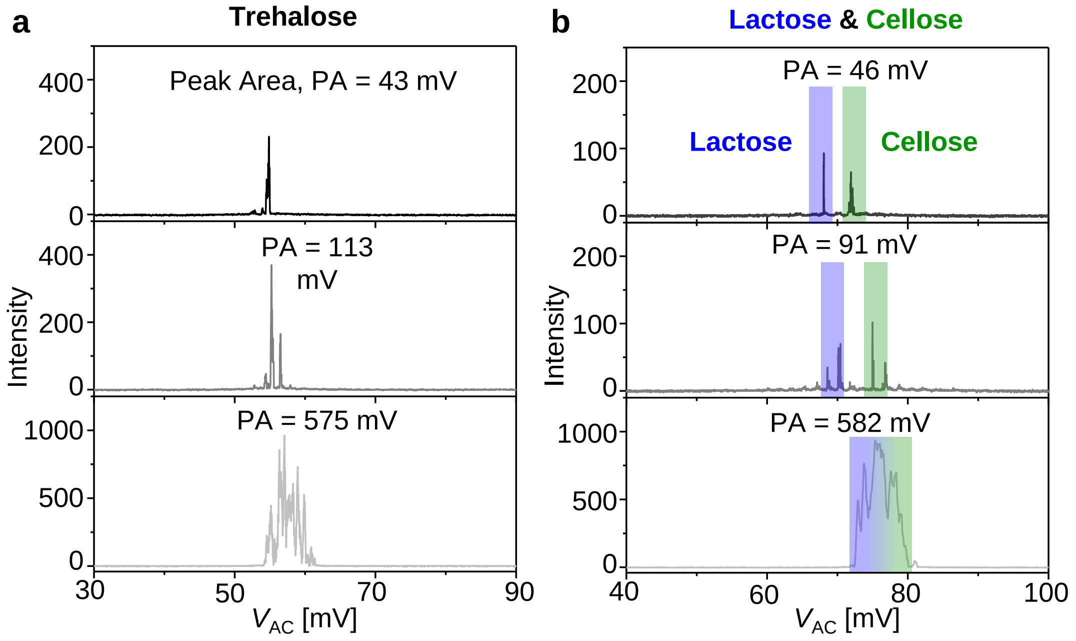

Supplementary Fig. 6. Ion cloud profiling spectra of disaccharide subjected to space charge effect. (**a**) The spectra of pure trehalose subjected to different space charge effects: PA = 43 mV (top), 113 mV (middle), 575 mV (bottom). Here, PA is the peak area of the ions in the spectrum. (**b**) The spectra of lactose and cellose mixture subjected to different space charge effects: PA = 46 mV (top), 91 mV (middle), 582 mV (bottom). The lactose and cellose peaks are marked by blue and green, respectively. Space charge effect due to the trapping of an excessive number of ions, e.g., the middle and bottom panels here, leads to performance degradation for structural analysis, such as the V_{AC} shift and peak splitting. In this work, as shown in top, ion number used for ion cloud profiling was optimized to ensure that each isomeric species generates a high-quality single peak in the spectrum, correspondingly.

When the ion number was optimized, performance of ion cloud profiling could be further optimized via tuning working parameters, such as the AC resonance frequency, gas pressure, and Mathieu parameter, q (Supplementary Fig. 7). As a common knowledge, better resolutions could be achieved with longer time for analysis. A unique feature of the ion trap used here is its infinite time for ion trapping, which could be used for the structural analysis of the isomers. By increasing the analysis time via decreasing the scan speed, a structural resolution

over 10 k were obtained for all the three different biomolecules (Supplementary Fig. 8).

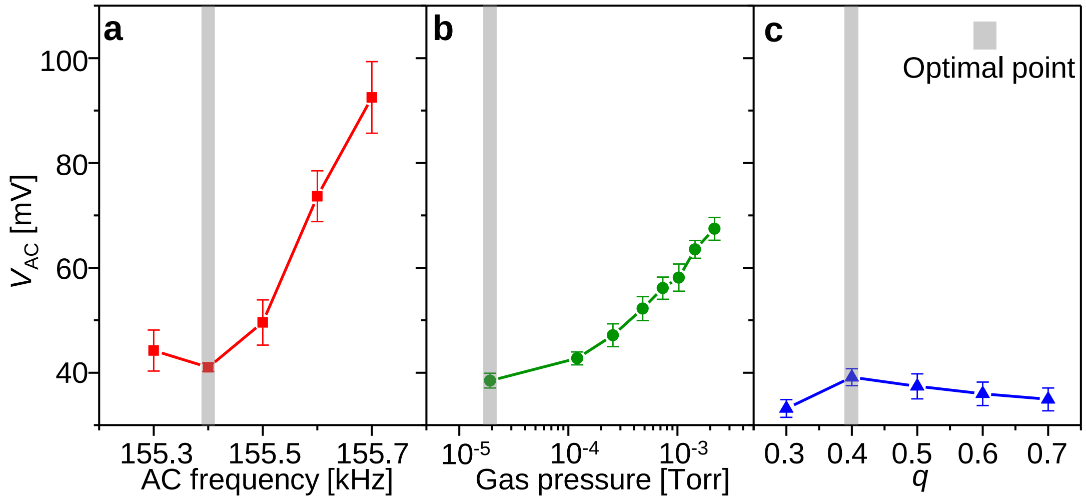

Supplementary Fig. 7. Parameter optimization for ion cloud profiling. Measured V_{AC} values as a function of (**a**) AC frequency, (**b**) gas pressure, (**c**) Mathieu parameter, q. At resonance, the V_{AC} has the lowest amplitude, which is used to identify the resonance frequency of the ions and AC. Optimal conditions are used by minimizing the uncertainty of the V_{AC}, as marked by the gray region. Error bar stands for one standard deviation for 10 replicates and centre of error bar is the mean of these replicates. Source data are provided as a Source Data file.

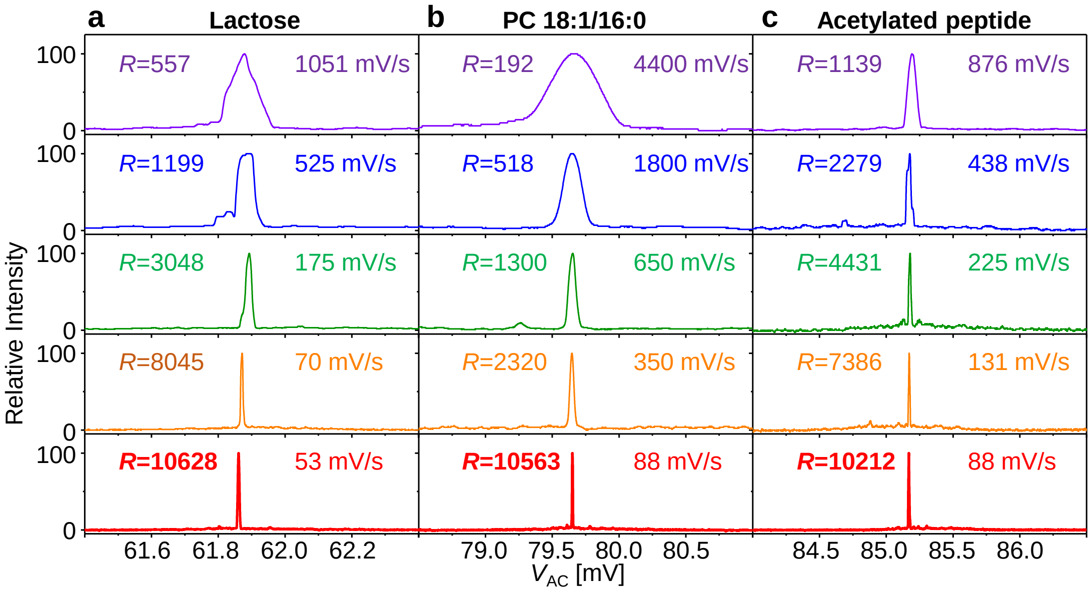

Supplementary Fig. 8. Ion cloud profiling spectra of three biomolecules of different V_{AC} scan rate. (**a**) The spectra of lactose at different scan rate: 1051

mV/s (violet), 525 mV/s (blue), 175 mV/s (green), 70 mV/s (orange), 53 mV/s (red). Here, scan rate is the scanned AC amplitude divided by the scan time. (**b**) The spectra of PC 18:1/16:0 at different scan rates: 4400 mV/s (violet), 1800 mV/s (blue), 650 mV/s (green), 350 mV/s (orange), 88 mV/s (red). (**c**) The spectra of acetylated peptide at different scan rates: 876 mV/s (violet), 438 mV/s (blue), 225 mV/s (green), 131 mV/s (orange), 88 mV/s (red). The peak resolutions were improved by decreasing the scan rate (from top to bottom).

For dynamic RF effect, the V_{AC} in each individual measurement might change slightly due to the uncertainty of the initial RF phase for ion analysis (Supplementary Fig. 9a). This resulted in a normal distribution of V_{AC} of during the measurements, e.g., the lactose (blue) and cellose (green) in Supplementary Fig. 9b. The uncertainty of V_{AC} due to the initial RF could be removed by using an averaged V_{AC} of replicate measurements, as shown in Supplementary Fig. 9c. Here, it should be note that the uncertainty of V_{AC} won't be a problem for mixture analysis because the V_{AC} shift of the mixture in one measurement were synchronized (Supplementary Fig. 10a). For lactose and cellose mixture, it was observed that their difference of V_{AC}, characterized by parameter d, kept stable in 10 replicate measurements (Supplementary Fig. 10b).

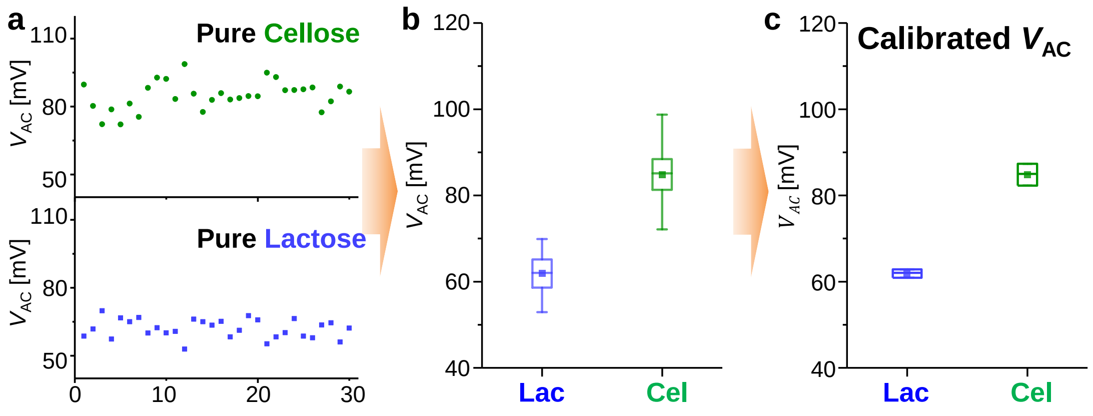

Supplementary Fig. 9. Calibration of V_{AC} values of two pure disaccharides. (**a**) V_{AC} values of pure cellose (green) and lactose (blue) for 30 replicates. (**b**) The V_{AC} distributions of lactose (blue) and cellose (green) shown in (**a**). (**c**) The distributions of V_{AC} mean values of lactose (blue) and cellose (green), represented by the blue and green square icons in (**b**) for 3 replicates. In this work, the V_{AC} values were calibrated to their

mean values shown in (**c**). Box plots show the mean (square icon), the median (horizontal line), interquartile range (hinges) and smallest and largest values no more than 1.5 times the interquartile range (whiskers). Source data are provided as a Source Data file.

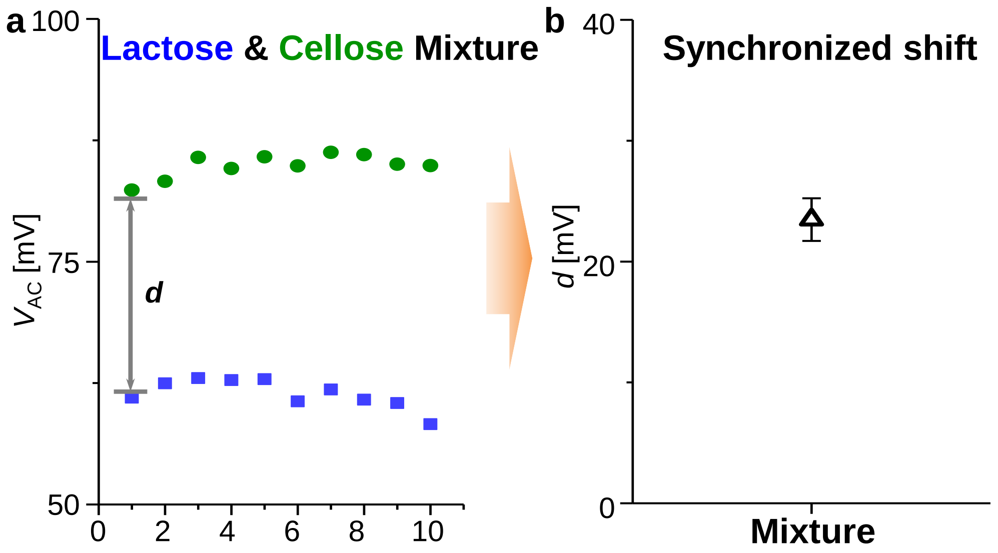

Supplementary Fig. 10. Synchronized shift of V_{AC} values of disaccharides mixture. (**a**) V_{AC} values of cellose (green) and lactose (blue) mixture for 10 replicates. The difference of V_{AC} values between lactose and cellose is defined as *d*. (**b**) The distribution of *d* shows synchronized shift of V_{AC} value of mixture during the ion cloud profiling. Error bar stands for one standard deviation of the distribution for 10 replicates and centre of error bar is the mean of these replicates. Source data are provided as a Source Data file.

Supplementary Note 4
Theoretical modelling

The model considered here described the ion motion in a LIT [17]. An electric potential, ϕ, was used for ion trapping, which had

$$\phi = \left(\frac{x^2-y^2}{r_0^2}\right)\ (U - V_{RF}\cos\Omega t) \qquad \text{(S1)}$$

Here, U stands for the direct current (DC) component and has $U = 0$ in this work. V_{RF} stands for the RF component at a secular frequency, $\Omega = 2\pi \times 1$ MHz.

The DC and RF components of ϕ could be characterized by Mathieu parameters, a_u and q_u:

$$a_x = -a_y = \frac{8eU}{m\Omega^2 r_0^2};\ q_x = -q_y = \frac{4eV_{RF}}{m\Omega^2 r_0^2} \quad \text{(S2)}$$

where m is ion mass, e is electron charge carried by the ions, u represents either x or y coordinates.

For ions trapped within the LIT, the equation of ion motion in the electric field was as following:

$$m\frac{d^2u}{dt^2} + b\frac{du}{dt} + e\nabla_u\phi = 0 \quad \text{(S3)}$$

Here, m is ion mass, e is electron charge carried by the ions, and b is damping coefficient of the ions.

For ions subjected to the AC resonance excitation in ion cloud profiling, the equation of ion motion became

$$m\frac{d^2u}{dt^2} + b\frac{du}{dt} + e\nabla_u\phi = C\sin(\omega t) \quad \text{(S4)}$$

Here, C represents the excitation strength and has $C = \alpha V_{AC}/2r_0$. V_{AC} is AC voltage with an angular frequency ω, α is calibration coefficient and has $\alpha \approx 0.8$ in this work.

To understand the ion cloud profiling theoretically, Equation S4 was further simplified by using pseudopotential well approximation [18], where the RF component V_{RF} could be approximately represented by an effective DC field, V_{eff}. Then, the effective electric potential, ϕ_{eff}, yields

$$\phi_{eff} = \left(\frac{x^2+y^2}{r_0^2}\right)\ V_{eff} \quad \text{(S5)}$$

Taking Equation S5 into Equation S4, the Equation S4 becomes the Equation 1 used in the main text.

Numerical simulation

Numerical simulation of Equation S4 was also performed by using the fourth-order Runge-Kutta method implemented in a home-made algorithm package, electro-hydrodynamic simulation (EHS) [19,20]. Two simulation ion species of disaccharide isomers, I: m/z = 365 and b' = 0.0010 (blue) and II: m/z = 365 and b' = 0.0012 (purple), were used to simulate the ion cloud profiling process under the AC excitation, V_{AC}, of 70 mV (Fig. 1c and Supplementary Fig. 2b). Here, $b' = 2b/\Omega m$, where Ω is the angular frequence of the RF field, m is ion mass, b is the damping coefficient of the ions. Each isomeric species had 100 simulation ions to simulate the motion behavior of ion cloud. The initial positions and velocities of the ions were sampled stochastically based on a thermal distribution at 300 K (Supplementary Fig. 2a). Four simulation ion species of disaccharide isomers analyzed in the experiment were used to simulate the ion cloud profiling process as well (Supplementary Fig. 3b). The simulation parameters of the LIT for ion manipulation were the same as disaccharide experiments shown in Supplementary Tables 2 and 3.

Supplementary References

1 Hofmann, J., Hahm, H. S., Seeberger, P. H. & Pagel, K. Identification of carbohydrate anomers using ion mobility–mass spectrometry. *Nature* **526**, 241-244 (2015).

2 Fouque, K. J. D. *et al.* Effective Liquid Chromatography-Trapped Ion Mobility Spectrometry-Mass Spectrometry Separation of Isomeric Lipid Species. *Anal. Chem.* **91**, 5021-5027 (2019).

3 Giles, K. *et al.* A Cyclic Ion Mobility-Mass Spectrometry System. *Anal. Chem.* **91**, 8564-8573 (2019).

4 Hollerbach, A. L. *et al.* Ultra-High-Resolution Ion Mobility Separations Over Extended Path Lengths and Mobility Ranges Achieved using a Multilevel Structures for Lossless Ion Manipulations Module. *Anal. Chem.* **92**, 7972-7979 (2020).

5 Deng, L. L. *et al.* Serpentine Ultralong Path with Extended Routing (SUPER) High Resolution Traveling Wave Ion Mobility-MS using Structures for Lossless Ion Manipulations. *Anal. Chem.* **89**, 4628-4634 (2017).

6 Cleven, C. D., Cooks, R. G., Garrett, A. W., Nogar, N. S. & Hemberger, P. H. Radial distributions and ejection times of molecular ions in an ion trap mass spectrometer: A laser tomography study of effects of ion density and molecular type. *J. Phys. Chem.* **100**, 40-46 (1996).

7 Kirk, A. T., Raddatz, C.-R. & Zimmermann, S. Separation of Isotopologues in Ultra-High-Resolution Ion Mobility Spectrometry. *Anal. Chem.* **89**, 1509–1515 (2017).

8 Kirk, A. T., Grube, D., Kobelt, T., Wendt, C. & Zimmermann, S. High-Resolution High Kinetic Energy Ion Mobility Spectrometer Based on a Low-Discrimination Tristate Ion Shutter. *Anal. Chem.* **90**, 5603–5611 (2018).

9 Glaskin, R. S., Ewing, M. A. & Clemmer, D. E. Ion Trapping for Ion Mobility Spectrometry Measurements in a Cyclical Drift Tube. *Anal. Chem.* **85**, 7003–7008 (2013).

10 Adams, K. J., Montero, D., Aga, D. & Fernandez-Lima, F. Isomer separation of polybrominated diphenyl ether metabolites using nanoESI-TIMS-MS. *Int. J. Ion Mobil. Spectrom.* **19**, 69–76 (2016).

11 Shvartsburg, A. A. *et al*. High-Definition Differential Ion Mobility Spectrometry with Resolving Power up to 500. *J. Am. Soc. Mass Spectrom.* **24**, 109–114 (2013).

12 Bythell, B. J., Abutokaikah, M. T., Wagoner, A. R., Guan, S. & Rabus, J. M.. Cationized Carbohydrate Gas-Phase Fragmentation Chemistry. *Journal of the American Society for Mass Spectrometry* **28,** 688–703 (2017).

13 May, J. C. *et al*. Conformational landscapes of ubiquitin, cytochrome c, and myoglobin: Uniform field ion mobility measurements in helium and nitrogen drift gas. *Int. J. Mass. Spec.* **427**, 79–90 (2018).

14 Zhou, X., Liu, X. & Ouyang, Z. Statistical Algorithm Enables Rapid Computation of Space Charge Effect and Spectral Correction in a Miniature Ion Trap Mass Spectrometer. *J. Am. Soc. Mass Spectrom.* **31**, 429–433 (2020)..

15 Allen, S. J. & Bush, M. F. Radio-Frequency (rf) Confinement in Ion Mobility Spectrometry: Apparent Mobilities and Effective Temperatures. *J. Am. Soc. Mass Spectrom.* **27**, 2054–2063 (2016).

16 Xiong, C. *et al*. A Theoretical Method for Characterizing Nonlinear Effects in Paul Traps with Added Octopole Field. *J. Am. Soc. Mass Spectrom.* **26**, 1338–1348 (2015). .

17 Nolting, D., Malek, R. & Makarov, A. Ion traps in modern mass spectrometry. *Mass Spectrom. Rev.* **38**, 150–168 (2019).

18 Dehmelt, H. G. Radiofrequency Spectroscopy of Stored Ions I: Storage. *Adv. At. Mol. Phys.* **3**, 53-72 (1968).

19 Zhou, X., Ouyang, Z. Flowing gas in mass spectrometer: method for characterization and impact on ion processing. *The Analyst* **139**, 5215-5222 (2014).

20 Zhou, X., Ouyang, Z. Following the Ions through a Mass Spectrometer with Atmospheric Pressure Interface: Simulation of Complete Ion Trajectories from Ion Source to Mass Analyzer. *Anal. Chem.* **88**, 7033-7040 (2016).
