# Supplementary material for: High-resolution separation of bioisomers using ion cloud profiling
Source: Nat Commun. 2023 Mar 20;14:1535. doi: 10.1038/s41467-023-37281-7 (PMC10027677; doi:10.1038/s41467-023-37281-7)
Supplement: Supplementary file 3 — Reporting Summary [file 41467_2023_37281_MOESM3_ESM.pdf]

nature portfolio

Corresponding author(s): Zheng Ouyang

Last updated by author(s): Feb 22, 2023

Reporting Summary

Nature Portfolio wishes to improve the reproducibility of the work that we publish. This form provides structure for consistency and transparency in reporting. For further information on Nature Portfolio policies, see our Editorial Policies and the Editorial Policy Checklist.

Statistics

For all statistical analyses, confirm that the following items are present in the figure legend, table legend, main text, or Methods section.

n/a	Confirmed	
☐	☒	The exact sample size (*n*) for each experimental group/condition, given as a discrete number and unit of measurement
☐	☒	A statement on whether measurements were taken from distinct samples or whether the same sample was measured repeatedly
☒	☐	The statistical test(s) used AND whether they are one- or two-sided<br>*Only common tests should be described solely by name; describe more complex techniques in the Methods section.*
☒	☐	A description of all covariates tested
☒	☐	A description of any assumptions or corrections, such as tests of normality and adjustment for multiple comparisons
☐	☒	A full description of the statistical parameters including central tendency (e.g. means) or other basic estimates (e.g. regression coefficient) AND variation (e.g. standard deviation) or associated estimates of uncertainty (e.g. confidence intervals)
☒	☐	For null hypothesis testing, the test statistic (e.g. *F*, *t*, *r*) with confidence intervals, effect sizes, degrees of freedom and *P* value noted<br>*Give P values as exact values whenever suitable.*
☒	☐	For Bayesian analysis, information on the choice of priors and Markov chain Monte Carlo settings
☒	☐	For hierarchical and complex designs, identification of the appropriate level for tests and full reporting of outcomes
☒	☐	Estimates of effect sizes (e.g. Cohen's *d*, Pearson's *r*), indicating how they were calculated

Our web collection on statistics for biologists contains articles on many of the points above.

Software and code

Policy information about availability of computer code

Data collection	SpecMS from PURSPEC
Data analysis	MATLAB 2017b from MathWorks

For manuscripts utilizing custom algorithms or software that are central to the research but not yet described in published literature, software must be made available to editors and reviewers. We strongly encourage code deposition in a community repository (e.g. GitHub). See the Nature Portfolio guidelines for submitting code & software for further information.

Data

Policy information about availability of data

All manuscripts must include a data availability statement. This statement should provide the following information, where applicable:

- Accession codes, unique identifiers, or web links for publicly available datasets
- A description of any restrictions on data availability
- For clinical datasets or third party data, please ensure that the statement adheres to our policy

A reporting summary for this Article is available as a Supplementary Information file.
All data supporting the findings of this study are available from the corresponding authors upon reasonable request.
The source data for Figs. 2e-f and supplementary Figs. 7, 9, 10 are provided in a Source Data file.
The raw data are available from Figshare (https://figshare.com/articles/dataset/Raw_data_of_High-

Resolution_Separation_of_Bioisomers_Using_Ion_Cloud_Profiling/22139816).

Human research participants

Policy information about studies involving human research participants and Sex and Gender in Research.

Reporting on sex and gender	Not relevant. The study didn't involve human research participants in research.
Population characteristics	Not relevant.
Recruitment	Not relevant.
Ethics oversight	Not relevant.

Note that full information on the approval of the study protocol must also be provided in the manuscript.

Field-specific reporting

Please select the one below that is the best fit for your research. If you are not sure, read the appropriate sections before making your selection.

[x] Life sciences [ ] Behavioural & social sciences [ ] Ecological, evolutionary & environmental sciences

For a reference copy of the document with all sections, see nature.com/documents/nr-reporting-summary-flat.pdf

Life sciences study design

All studies must disclose on these points even when the disclosure is negative.

Sample size	We used 4 kinds of disaccharides, 5 kinds of phospholipids, 6 kinds of peptides SGKLRASHKG with methylation in K3 or K9, acetylation in K3 or K9, and phosphorylation in S1 or S7. These samples cover the basic isomerism types in disaccharides, phospholipids, and peptides with post-translational modifications. No sample size calculation was performed and the sample size showed a relative reproducibility of isomer structure analysis results.
Data exclusions	No data were excluded.
Replication	For quantitative analysis of pure sample, 10 independent experiments were performed with identical results.<br>For quantitative analysis of mixture, 15 independent experiments were performed with identical results.<br>For parameter optimization for ion cloud profiling, 10 independent experiments were performed with identical results.<br>For calibration of VAC values of pure disaccharides, 30 independent experiments were performed with identical results.<br>For calibration of VAC values of pure disaccharides, 10 independent experiments were performed with identical results.
Randomization	The disaccharides were groups according to 3 kinds of isomerization including composition, connectivity and configuration. The phospholipids were groups according to 3 kinds of isomerization including sn- positions of the fatty acyl chains, C=C locations and configurations. The peptides with post-translational modifications were groups according to 3 kinds modified group of including methylation acetylation and phosphorylation.
Blinding	Blinding was not applied, because the demonstrations in different isomers were just to show the differences among these isomers.

Reporting for specific materials, systems and methods

We require information from authors about some types of materials, experimental systems and methods used in many studies. Here, indicate whether each material, system or method listed is relevant to your study. If you are not sure if a list item applies to your research, read the appropriate section before selecting a response.

Materials & experimental systems

n/a	Involved in the study
[x]	[ ] Antibodies
[x]	[ ] Eukaryotic cell lines
[x]	[ ] Palaeontology and archaeology
[x]	[ ] Animals and other organisms
[x]	[ ] Clinical data
[x]	[ ] Dual use research of concern

Methods

n/a	Involved in the study
[x]	[ ] ChIP-seq
[x]	[ ] Flow cytometry
[x]	[ ] MRI-based neuroimaging
